# Protocol for a prospective multicenter cross-sectional observational study to investigate the role of air pollution on allergic rhinitis prevalence

**DOI:** 10.1097/MD.0000000000019497

**Published:** 2020-03-13

**Authors:** Jianmin Liu, Yongkuan Wang, Sisi Liu, Shuwei Cao, Chunyan Xu, Meng Zhang, Shixi Liu

**Affiliations:** a Department of Otolaryngology, Head and Neck Surgery, West China Hospital, Sichuan University, Chengdu, Sichuan; b Department of Otolaryngology, Head and Neck Surgery, People's Hospital of De Yang City, De Yang; c Jane Lab, Big Data Research Center, University of Electronic Science and Technology of China, Chengdu, China.

**Keywords:** allergic rhinitis, air pollution, NO_2_, SO_2_, PM10, PM2.5

## Abstract

**Introduction:**

Allergic rhinitis (AR) is a major chronic inflammatory disease of the respiratory tract. A large number of epidemiological investigations have shown that the prevalence of AR is increasing, resulting in a large social burden. Importantly, the impact of air pollution on health is a widespread concern. We aim to evaluate association of air pollution and AR risk.

**Methods and analysis:**

This prospective study includes patients undergoing AR. The exclusion criteria will be as follows: Patients with nasal infection, nasal polyps, nasal tumors, mental disorders, and immunodeficiency will be excluded. Air pollution levels of ambient air pollutants including PM2.5, PM10, sulfur dioxide (SO_2_), nitrogen dioxide (NO_2_), carbon dioxide (CO), and O_3_, and patient data will be collected. The correlation analysis will be performed in air pollutants and AR risk.

**Discussion:**

This study will provide correlation of NO_2_, SO_2_, PM10, and PM2.5 for AR in several aspects, including symptom score, drug score, quality of life score, asthma control score, side effects, and laboratory examination such as nasal function test, serum total immunoglobulin E, and nasal secretion smear.

## 1 Introduction

Rapid economic development and the acceleration of urbanization have aggravated the haze in Sichuan, including Deyang city, an important industrial city in north Sichuan. The impact of air pollution exposure on human health has become a sensitive topic for the public, media, and even the government in China and neighboring countries.^[1]^ To evaluate the impact of outdoor air pollution on human health and implement appropriate protection policies, relevant studies have been conducted in the past decades.^[2,3]^ Many adverse health problems have been reported associated with air pollution, including respiratory diseases (rhinitis, asthma, bronchitis, and pneumonia),^[4]^ cardiovascular diseases (stroke, cardiac arrhythmias, and ischemic heart disease),^[5]^ chronic obstructive pulmonary disease (COPD),^[6]^ more rarely, conjunctivitis,^[7]^ skin disease,^[8]^ and cough.^[9]^ Researches on the correlation between air pollution and respiratory diseases (such as asthma and COPD) are abundant,^[6]^ while few studies investigated the correlation of allergic rhinitis (AR) with air pollution in China.

The AR is a noninfectious respiratory inflammatory disease of nasal mucosa mediated by immunoglobulin E.^[10]^ Moreover, AR involves a variety of immune-active cells and cytokines after atopic individuals are exposed to allergens, affecting 10% to 40% in the world.^[11]^ In China, the prevalence rate of adults is about 4% to 38%.^[12]^ Due to the easy relapse of AR and the delay in the course of the disease, the life, and work of the patients has been seriously affected. Some AR patients have suffered from asthma,^[13]^ nasal polyps, and rhinosinusitis.^[14]^ Obviously, AR has become a public health problem. Some experts believe that the increase in the prevalence of AR is caused by the increase of air pollutants^[15–19]^; however, there is still no hard evidence to support it. At present, there are no continuous and dynamic studies to observe the changes of pollutants and the AR risk.

This study was designed to assess the relationship between air pollution exposure and the risk of AR.

## 2 Methods and analysis

### 2.1 Main aims

**Figure 1:**
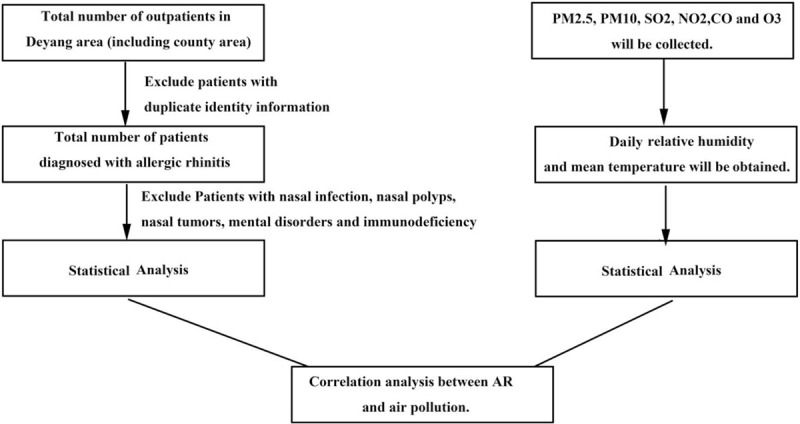
Flow chart of study protocol. AR = allergic rhinitis, CO = carbon dioxide.

We aim to clarify the relationship between air pollution exposure and the risk of AR. Flow chart of study protocol is shown in Figure 1.

#### 2.1.1 Special aim

Aim 1: By collecting the general clinical data of patients with AR in multicenter outpatient clinics in Deyang region from January 2019 to December 2020, the prevalence of AR in outpatient clinics in Deyang region was investigated.

Aim 2: To collect air pollution indicators including PM2.5, PM10, NO_2_, and SO_2_ in Deyang area and discuss the correlation between pollutants and the prevalence of AR.

Aim 3: To understand the quality of life of AR patients in the Asia-Pacific region via analyzing the correlation between AR symptoms and atmospheric pollutants.

Aim 4: To provide targeted guidance and Suggestions for government departments to prevent airway inflammation and other diseases caused by air pollution. In this way, medical expenses of the public and health departments can be reduced and the quality of life of patients can be improved.

### 2.2 Study registration

The protocol scheme matches PRISMA's reporting standards. This study protocol has been registered on Chinese Clinical trial registry (http://www.chictr.org.cn/index.aspx) with a unique ID of ChiCTR2000029027.

### 2.3 Participants

All age groups (newborn to adult) suffering from any form of AR will be included. These may include participants with acute or chronic comorbidities but without immunodeficiency. Participants may also include those on conventional treatment for other health issues. Symptoms of AR include rhinorrhea, nasal obstruction, nasal itching, and sneezing which are reversible spontaneously or with treatment. A diagnosis of AR will include the following symptoms based on the Guideline Summary of Management of Allergic and Non-AR: Nose: nasal discharge (runny nose), sneezing, nasal blockage or congestion, and itchy nose and palate; Eye: bilateral itchy eyes, red eyes (concomitant allergic conjunctivitis), and swollen eyes.

There is no specific duration of symptoms required for diagnosis; however, a very short or nonrepetitive history of symptoms generally excludes a diagnosis of AR. The presence of any one of the nasal symptoms will be essential for a diagnosis of AR except when nasal blockage is the only symptom. Nasal blockage on its own rarely indicates allergy. The duration of symptoms will not be present as a criterion since this has not been defined for a diagnosis of AR.

#### 2.3.1 Inclusion criteria

All participants will be diagnosed as AR in accordance with the diagnosis and treatment guidelines for AR (2015, Tianjin), and will be diagnosed by a senior attending physician who had received unified training. All the patients participating in the questionnaire can understand the questionnaire accurately and cooperate with the successful completion of the questionnaire survey.

#### 2.3.2 Exclusion criteria

The exclusion criteria are as follows: Patients with nasal infection, nasal polyps, nasal tumors, mental disorders, and immunodeficiency will be excluded.

#### 2.3.3 Recruitment

Patient data will be collected for all patients with AR, including general data such as sex, age, sex, place of residence, course of disease, and main clinical symptoms.

### 2.4 Study areas

Deyang is the North city of Sichuan Province and closed to Sichuan capital city Chengdu. In recent years, PM pollution has become a serious issue for this city due to its large population and automobile and industrial emissions.

### 2.5 Data collection

Each area in Deyang city (including Jingyang, Shifang, and Mianzhu city of Sichuan Guanghan, Luojiang district, and Zhongjiang county) outpatient department of otolaryngology head and neck surgery patients, according to the guide to conform to the diagnosis and treatment of AR (2015, Tianjin) for inclusion criteria, the general clinical data (including gender, age, duration, attack frequency, main symptom, etc) each year, Deyang area in patients with AR disease registration rates.

Air pollution levels of ambient air pollutants including PM2.5, PM10, sulfur dioxide (SO_2_), nitrogen dioxide (NO_2_), carbon dioxide (CO), and O_3_ will be derived from the China National Environmental Monitoring Center (http://www.cnemc.cn/). Daily data of weather conditions, including relative humidity (%) and mean temperature (°C), will be obtained from the China Meteorological Monitoring Database (http://data.cma.cn/).

### 2.6 Statistical plan

Continuous variables will be expressed as mean ± standard deviation, while nominal variables are expressed in frequency and percentage. Comparisons of continuous variables will be performed using the 2-tailed *t* test for unpaired samples or Welch *t* test as appropriate. Comparisons of nominal variables will be performed using the Chi-squared test or Fisher exact test as appropriate. Multivariate logistic regression test will be used to evaluate the association between predictor variables and outcome variables. Statistical significance will be defined by a *P* value of <.05. The statistics will be performed using SAS 9.4 (SAS Institute Inc, Cary, NC).

The data used for the study will be preserved and analyzed by the primary investigator. The data are accessible only to the primary investigator and study nurse for data safety. The data will be preserved for 2 years after the end of the study.

### 2.7 Ethics and dissemination

This study has been approved by the ethics committee of Deyang People's Hospital. All participants will sign the informed consent after being informed about the goals and methods of the study. The present study will be conducted in accordance with the tenets of the 1975 Declaration of Helsinki, as revised in 2000. The result of the study will be disseminated by publication as journal articles.

## 3 Discussion

Globally, studies have shown an association between automotive and industrial emissions and increased AR risk.^[20]^ Individual pollutants are responsible for increased risk of allergic disease in nitrogen oxides (NOx), SO_2_, O_3_, particulate aerodynamic diameters of 10 μm or less (PM10), and particulate aerodynamic diameters of 2.5 μm or less (PM2.5).^[12]^ However, for environmental pollutants, only 6 studies have reported the effects of outdoor air pollutants and the prevalence of AR in the Asian atmosphere.

A national cross-sectional study covering Taiwanese school children showed that AR prevalence was associated with SO_2_, CO, and NOx levels, but not O_3_ and PM10 levels.^[21]^ Another Taiwanese study reported that AR in children is associated with nonsummer warmth and traffic-related air pollutant levels, including CO, NOx, and O_3_.^[22]^ For Asian adults, a cross-sectional population study in Singapore found that outdoor air pollution is an important environmental risk factor for AR.^[23]^ During 2007 to 2008, the concentrations of NO_2_ and SO_2_ were associated with an increase in the prevalence of AR among kindergarten children in 7 cities in Liaoning (China), but not with PM10.^[24]^ In addition, a study in Changsha (China) showed a significant positive correlation between the prevalence of AR in children and cumulative individual exposure to age-related PM10, SO_2_, and NO_2_.^[25]^ In this protocol, we will pay more attention on the effects of air pollutants (especially particulate matter) on AR in Deyang, Sichuan.

## Author contributions

XXX.

Meng Zhang orcid: 0000-0002-3731-7816.
